# Coccidioidomycosis Exposure Assessed by Skin Testing and Environmental Factors in Baja California, Mexico

**DOI:** 10.3390/jof12060407

**Published:** 2026-06-03

**Authors:** Ofelia Candolfi-Arballo, Amanda Dávila-Lezama, Erik Narváez-Hernández, Manuel Ontiveros-Duries, Jesús Manuel Soto-Reyes, José Mauricio Galeana-Pizaña, Nydia Alejandra Castillo-Martínez, Laura Rosio Castañón-Olivares

**Affiliations:** 1 Faculty of Health Sciences, Autonomous University of Baja California (UABC), Blvd Universitario No. 1000, Valle San Pedro, Tijuana 21500, Mexico; ocandolfi@uabc.edu.mx (O.C.-A.); adavila@uabc.edu.mx (A.D.-L.); erik.narvaez@uabc.edu.mx (E.N.-H.); manuel.ontiveros@uabc.edu.mx (M.O.-D.); jesus.manuel.soto.reyes@uabc.edu.mx (J.M.S.-R.); 2 Mexican Social Security Institute (IMSS), Calle Primera No. 500, Maclovio Herrera, Tecate 21482, Mexico; 3 Center for Geospatial Information Sciences (CentroGeo), Contoy No. 137, Tlalpan, Mexico City 14240, Mexico; mgaleana@centrogeo.edu.mx; 4 Mycology Unit, Department of Microbiology, Faculty of Medicine, National Autonomous University of Mexico (UNAM), Avenida Universidad No. 3000, Coyoacán, Mexico City 04510, Mexico

**Keywords:** coccidioidomycosis, skin tests, environmental exposure, epidemiology, Baja California, Mexico

## Abstract

Baja California is the second-highest state in Mexico for hospital discharges attributed to coccidioidomycosis (CM), yet epidemiological information on exposure patterns in affected communities remains limited. To estimate exposure to *Coccidioides* and assess its association with environmental factors, we conducted intradermal coccidioidin skin testing among 416 residents across nine regions of Baja California. We analyzed 24 environmental variables, including bioclimatic, topographic, and land use indicators. Overall, 31.9% of participants tested positive. Higher odds of exposure were observed in Valle de las Palmas and La Morita. Exploratory comparisons of environmental variables showed that, in unadjusted analyses, annual precipitation, precipitation during the wettest month, and elevation differed between high- and low-positivity localities. However, after applying the Benjamini–Hochberg false discovery rate correction, none of the evaluated continuous environmental variables remained statistically significant. These findings should therefore be interpreted as exploratory and hypothesis-generating rather than as evidence of an independently defined environmental profile. Overall, the results indicate heterogeneous exposure to *Coccidioides* across Baja California and suggest exploratory spatial variability in exposure across sampled localities. Because participants were recruited through nonprobability community-based sampling, these findings should be interpreted as exploratory rather than population-representative estimates.

## 1. Introduction

Coccidioidomycosis (CM) is an infection caused by fungi of the genus *Coccidioides*, characterized by a wide clinical spectrum ranging from asymptomatic disease to severe, life-threatening illness. This disease is contracted by inhaling fungal spores found in contaminated dust or air. The incubation period lasts 1 to 3 weeks, and although the infection is usually self-limiting, when clinical symptoms do appear, they can range from mild—such as fever or cough—to severe—such as shortness of breath, pneumonia, or even disseminated disease, in which case it can be fatal [1,2,3]. These clinical manifestations occur in approximately 40% of infected individuals and most frequently affect the respiratory system, skin, soft tissues, bones, and the central nervous system. Although anyone can be exposed to the fungus, those with weakened immune systems are at higher risk of developing severe disease, including people with HIV, pregnant women, older adults, and children, among others [2,4,5]. Additionally, other common risk factors have been identified when there is coinfection of CM with other respiratory diseases, such as diabetes mellitus and smoking [6].

After the United States, Mexico has the second-highest number of reported human cases worldwide, with most cases concentrated in the states of Sonora and Baja California [7,8]. According to national surveillance records, Mexico documented 2425 hospital discharges for CM from 2013 through 2023, of which 338 (13.94%) occurred in Baja California [8]. This state is therefore the second-largest contributor to national case burden.

Historically, estimates of *Coccidioides* exposure in Mexico have relied on the intradermal skin test (ST) with coccidioidin or spherulin. A positive ST (ST+) indicates prior exposure to the antigen rather than active infection [9]. In Baja California, these tests have been reported almost exclusively in the city of Tijuana, where positivity rates of 10% were documented in 1991 [8], 27.5% in 2010 among the general population [10], and 10% in pediatric populations in 1999 [11]. However, these studies did not include other regions of the state, leaving substantial gaps in understanding the geographic distribution of exposure.

Environmental surveillance in 2012 [12] and 2015 [13] detected *Coccidioides* spp. DNA in soil samples from Valle de las Palmas using nested PCR targeting ITS regions, indicating that the fungus was present in that area at least transiently. In 2016, a coccidioidin-ST was conducted to assess exposure among populations with varying levels of exposure: (1) 273 university students who attended classes in Valle de las Palmas but lived elsewhere, among whom 18 (6.6%) tested positive [14]; and (2) 10 residents of Valle de las Palmas, of whom 6 (60%) were positive [15]. These findings suggest that duration and intensity of exposure may influence immune sensitization and ST reactivity.

Given the increasing number of CM cases in Baja California and the limited availability of statewide epidemiologic data on *Coccidioides* exposure across different communities and geographic settings, more comprehensive studies are necessary to better understand regional risk patterns. This study aimed to estimate exposure to *Coccidioides* in Baja California using the coccidioidin-ST and to evaluate the association of this exposure with environmental variables that influence fungal growth, survival, and dispersal.

## 2. Materials and Methods

### 2.1. Population and Sampling

Participants were recruited from nine regions of Baja California: Ensenada (Ensenada municipality), Mexicali (Mexicali), Plan Libertador (Rosarito), San Felipe (San Felipe), Vicente Guerrero (San Quintín), Valle de las Palmas (Tecate), and the communities of El Niño, Terrazas del Valle, and La Morita (Tijuana). A nonprobability sampling strategy was used. State health institutions collaborated by providing facilities and mobilizing residents for coccidioidin-ST. Surveys were conducted between 2016 and 2022. Each attendee received an explanation of the study objectives and was informed that individuals of any age, sex, or respiratory symptom status were eligible to participate. **Inclusion criteria included voluntary participation and provision of written informed consent.** Individuals with severe immunosuppression or undergoing chemotherapy at the time of recruitment were not eligible for ST application. **Exclusion criteria, therefore, included severe immunosuppression and current chemotherapy treatment.** Individuals who voluntarily agreed to participate provided written informed consent under approval by the National Bioethics Commission (CONBIOÉTICA-02-CEI-001-20170526) [16]. For minors, consent was obtained from a parent or legal guardian.

Sociodemographic data (age, sex, residence, travel history) and clinical information (current or chronic underlying conditions, respiratory symptoms within the past 3 months, history of prior skin testing, and any adverse reactions to coccidioidin-ST) were collected through structured interviews.

### 2.2. Antigen

Coccidioidin was produced by the Institute of Epidemiological Diagnosis and Reference (INDRE, Mexico City, Mexico) of the National Ministry of Health [17,18,19].

### 2.3. Application

Each participant received an intradermal injection of 0.1 mL of coccidioidin using a hypodermic syringe (BD, Franklin Lakes, NJ, USA). The antigen was administered into the mid-third of the volar surface of the nondominant forearm. After injection, participants were asked to remain on site for 15 min to monitor for immediate adverse effects.

### 2.4. Interpretation of Intradermal Test Results

All coccidioidin-ST applications and induration measurements were performed by previously trained evaluators following standardized interpretation criteria. To maintain consistency throughout the study period, the same production lot of coccidioidin antigen was used across all study sites and collection periods.

Coccidioidin-ST results were evaluated between 48 and 72 h after application. The injection site was assessed for reaction characteristics. The presence or absence of erythema was noted; if induration was present, it was measured in millimeters. A reaction with an induration diameter ≥ 5 mm was classified as positive (coccidioidin-ST+), indicating prior exposure to *Coccidioides*.

### 2.5. Environmental Parameters

Twenty-four variables were analyzed, including 19 bioclimatic indicators derived from CHELSA-BIOCLIM+ climate-related predictors, representing the projected climatic dynamics for the 2011–2040 period at approximately 1 km spatial resolution [20]; climate classification [21]; two topographic variables (slope and elevation) [22]; and predominant land use and vegetation [23] surrounding each locality. Localities were categorized into high- and low-positivity groups for exploratory environmental comparisons. Because environmental variables were summarized at the locality level and only nine localities were included, environmental analyses were considered exploratory and hypothesis-generating.

### 2.6. Statistical Analysis

Statistical analyses were performed using Stata version 18 (StataCorp LLC, College Station, TX, USA; RRID:SCR_012763). Categorical variables were compared using the chi-square test. Associations between locality and coccidioidin-ST+ status were evaluated by calculating odds ratios (ORs) with 95% confidence intervals in Stata. Multivariable logistic regression analyses were additionally performed to evaluate factors independently associated with coccidioidin-ST+ while adjusting for age and sex. Adjusted odds ratios (aORs) with 95% confidence intervals (95% CI) were estimated. Analyses were performed using complete-case analysis, and the final adjusted model included 369 participants with complete information for locality, age, sex, and coccidioidin-ST results. Differences in induration size across localities were additionally evaluated using the nonparametric Kruskal–Wallis test. Environmental comparisons between high- and low-positivity localities were conducted using the nonparametric Mann–Whitney U test implemented with the wilcox.test function in R version 4.5.2 (R Foundation for Statistical Computing, Vienna, Austria; RRID:SCR_001905). Because multiple continuous environmental variables were evaluated, *p*-values were adjusted using the Benjamini–Hochberg false discovery rate procedure.

## 3. Results

A total of 521 persons received coccidioidin-ST during 2016–2022; however, only 416 returned for test interpretation. The spatial distribution of the evaluated population was as follows: Ensenada (n = 11; 2.64%), Mexicali (106; 25.48%), Plan Libertador (37; 8.89%), San Felipe (30; 7.21%), Vicente Guerrero (74; 17.78%), La Morita (37; 8.89%), El Niño (11; 2.64%), Terrazas del Valle (47; 11.29%), and Valle de las Palmas (63; 15.14%).

**Figure 1:**
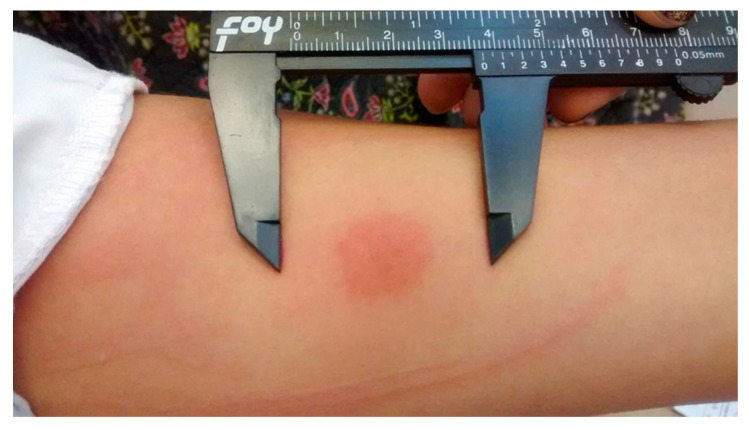
Coccidioidin-ST+ reaction showing a 36 mm induration.

Among participants, 66.2% were female and 33.8% male. Ages ranged from 6 months to 86 years (mean 38.5 years; median 37.5 years); nearly half (49%) were 19–40 years old. Most participants (85.6%) reported no travel to Sonora, California, and/or Arizona (USA). Domestic work was reported by 22.4%; 66.2% denied allergies; 47.9% reported no chronic underlying disease; and 58.7% had not experienced respiratory illness within the previous 3 months. Most tests were administered in 2019 (23.8%) and 2020 (32.6%). Overall, 31.9% (133/416) were coccidioidin-ST+; 41.3% had indurations 5–15 mm, and indurations > 20 mm were observed in all localities (Figure 1).

Bivariate analysis showed no significant associations between coccidioidin-ST+ and age, travel history, underlying diseases, or recent respiratory illness. Significant differences were observed in the absence of allergies, in the sampling years 2017 and 2019, and among persons reporting domestic work (Table 1).

**Figure 2:**
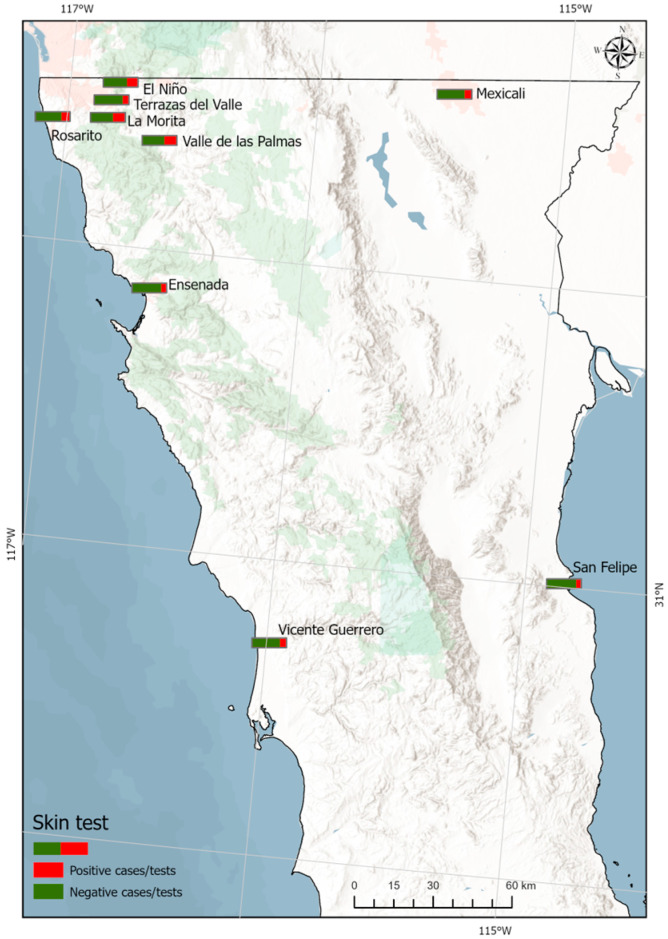
Exploratory distribution of coccidioidin-ST positive and negative results across sampled localities in Baja California, Mexico.

Because participant recruitment and sample sizes varied across localities, associations between coccidioidin-ST+ and locality should be interpreted as exploratory rather than population-representative comparisons (Figure 2). Using San Felipe (lowest positivity) as the reference group, higher odds of exposure to *Coccidioides* spp. were observed in Valle de las Palmas (OR 6.25, 95% CI 2.12–18.43; *p* = 0.001) and La Morita (OR 5.88, 95% CI 1.85–18.72; *p* = 0.003) (Table 2). Based on the most recent subnational survey in Mexico (2011), which reported an average positivity of 29.5% across endemic and non-endemic states [10], study localities were categorized as low-positivity (≤30%) or high-positivity (>30%), as shown in Table 2. In multivariable logistic regression analyses adjusted for age and sex, Valle de las Palmas (aOR 7.58, 95% CI 2.44–23.53; *p* < 0.001) and La Morita (aOR 7.22, 95% CI 2.18–23.90; *p* = 0.001) remained significantly associated with coccidioidin-ST+. El Niño and Plan Libertador also remained significantly associated after adjustment. Complete multivariable logistic regression results are presented in Supplementary Table S1. Additional exploratory models, including occupation, travel history, and chronic disease variables, were also evaluated; however, sparse categories, low-frequency strata, and missing data reduced the effective sample size and produced unstable estimates. Therefore, the more parsimonious model adjusted for age and sex was retained for the primary multivariable analysis. No significant associations were observed for age or sex after adjustment. Additionally, Kruskal–Wallis analyses demonstrated significant differences in induration size across localities (*p* < 0.001), suggesting heterogeneity in the magnitude of ST responses among study regions.

**Figure 3:**
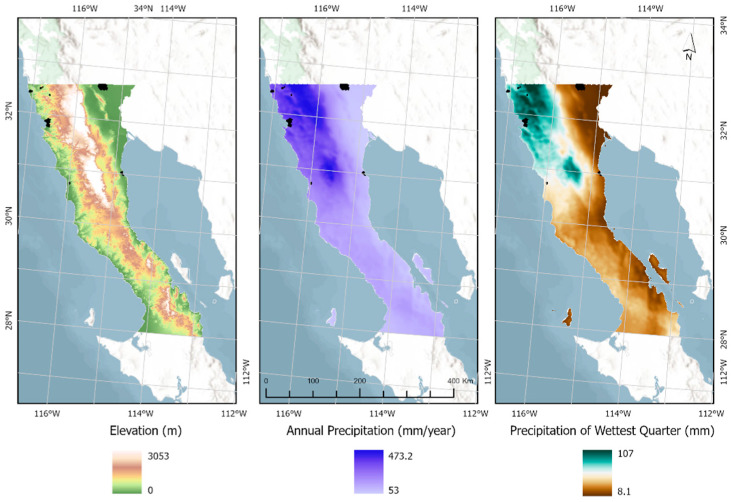
Spatial distribution of annual precipitation, precipitation during the wettest month, and elevation across the studied localities in Baja California, Mexico. These variables showed exploratory locality-level patterns between high- and low-positivity localities, but none remained statistically significant after Benjamini–Hochberg false discovery rate correction.

Environmental comparisons between high- and low-positivity groups showed the strongest unadjusted differences in three abiotic variables: annual precipitation, precipitation during the wettest month, and elevation. However, because multiple environmental variables were evaluated, *p*-values were subsequently adjusted using the Benjamini–Hochberg false discovery rate procedure. After correction, none of the evaluated continuous environmental variables remained statistically significant. Therefore, these findings were interpreted as exploratory locality-level environmental patterns rather than confirmatory associations. Detailed Mann–Whitney U test results, including adjusted *p*-values, are presented in Supplementary Table S2. Descriptive values for these variables across localities are shown in Supplementary Table S3. High-positivity localities showed annual precipitation values of 281.00–337.00 mm, wettest-month precipitation values of 52.70–63.40 mm, and elevations of 125.02–276.44 m above sea level. In contrast, lower-positivity localities such as Mexicali and San Felipe generally showed hotter, drier, and lower-elevation environmental conditions, whereas higher-positivity localities tended to occur in areas with relatively greater precipitation and intermediate elevations. Figure 3 illustrates the spatial distribution of annual precipitation, precipitation during the wettest month, and elevation across the sampled localities, providing geographic context for these exploratory environmental patterns.

## 4. Discussion

Among the 133 coccidioidin-ST+ individuals, a larger, although not statistically significant, proportion reported no underlying diseases or immunodeficiencies, suggesting that most participants likely maintained preserved immune function. This pattern is consistent with the interpretation that coccidioidin-ST+ reflects prior exposure, frequently associated with subclinical or asymptomatic infection [5]. However, some participants reported chronic conditions potentially associated with altered immune responses, including diabetes and HIV infection. Overall, 27% of the study population reported chronic underlying conditions, primarily diabetes and hypertension, which may alter delayed-type hypersensitivity responses and reduce the likelihood of detectable ST reactivity despite previous exposure to *Coccidioides* [24]. Negative coccidioidin-ST results should therefore be interpreted within the limitations of ST–based exposure assessment, because impaired cellular immune responses may result in false-negative reactions despite prior exposure. Because immune function was not directly evaluated, the relationship between chronic diseases and ST reactivity should also be interpreted within the exploratory context of the study. The detected positivity rates highlight the importance of strengthening community-based education, preventive measures, and environmental risk awareness in populations potentially exposed to *Coccidioides*.

The higher proportion of coccidioidin-ST+ observed among homemakers should not be interpreted as evidence of a direct occupational effect. Instead, this category may reflect indirect environmental exposure patterns associated with peri-urban housing conditions, unpaved surroundings, domestic outdoor activities, or locality-specific socioeconomic characteristics. Informal or intermittent labor activities may not have been fully captured by the occupational categories used in this study. Furthermore, occupation may correlate with locality-specific environmental and socioeconomic conditions that were not fully captured in the present analysis. The higher frequency of coccidioidin-ST+ among individuals who reported no travel to recognized endemic areas in Mexico or the United States is consistent with the possibility of local exposure within Baja California. Additionally, the higher proportion of coccidioidin-ST+ individuals without recent respiratory illness may reflect asymptomatic infection or the attribution of mild symptoms to other acute respiratory illnesses such as influenza or COVID-19. In 2024 alone, Baja California reported more than 400,000 respiratory cases [25], none of which underwent differential diagnosis for CM. Although 31.9% of participants in the present study showed evidence of prior exposure to *Coccidioides*, only 338 CM cases were officially reported in Baja California from 2013 to 2023. This discrepancy between the high proportion of coccidioidin-ST+ and the relatively low number of reported cases suggests that CM remains underrecognized and likely underdiagnosed in the region. Similar concerns about underrecognition and limited surveillance have been raised in other endemic areas [26].

Some significant associations, including higher coccidioidin-ST+ among participants without allergies and among specific sampling years or occupational groups, may reflect underlying recruitment patterns rather than true epidemiologic differences. These findings should therefore be interpreted within the limitations of the exploratory study design.

Baja California has a predominantly arid climate, with regional variability influenced by latitude, topography, altitude, and Pacific Ocean dynamics [27]. Although climate type did not differ between groups, the four localities with the highest positivity shared the BSks subtype (cold semi-arid steppe climate with winter precipitation). These areas were also characterized by induced or cultivated grasslands, which are atypical within the natural vegetation of Baja California. Nonetheless, these grasslands may create microenvironments that promote moisture retention and increase soil coverage, thereby potentially favoring environmental persistence of *Coccidioides*.

In this exploratory locality-level analysis, high-positivity localities descriptively tended to occur in areas with relatively greater precipitation and intermediate elevations. However, these patterns were based on only nine localities, and none of the evaluated continuous environmental variables remained statistically significant after correction for multiple testing. Therefore, these observations should be interpreted only as descriptive geographic context and not as evidence of environmental associations.

Although some descriptive characteristics of the sampled high-positivity localities, including semi-arid conditions, intermediate elevations, and relatively greater precipitation, are consistent with environmental features reported in endemic regions of the southwestern United States [28,29,30], the present analysis was not designed to identify environmental predictors. Given the small number of localities and the lack of statistically significant continuous environmental variables after multiple-testing correction, these comparisons should be considered background context for future studies rather than evidence of locality-level environmental determinants.

La Morita and El Niño, located on the eastern edge of Tijuana, are characterized by high population mobility, including migrant populations and maquiladora workers, as well as limited urban infrastructure and socioeconomic vulnerability [31]. Such conditions may increase exposure to dust and disturbed soil [32,33,34], potentially amplifying the risk of *Coccidioides* exposure in these communities. In multivariable logistic regression analyses adjusted for age and sex, the associations observed for Valle de las Palmas and La Morita remained statistically significant, remaining consistent with the exploratory geographic variability observed across the sampled localities. El Niño and Plan Libertador also remained significantly associated with coccidioidin-ST+ after adjustment, although these findings should be interpreted cautiously because of the exploratory nature of the study and the heterogeneity of the sampled populations. These findings highlight exploratory geographic variability in coccidioidin-ST+ among the sampled localities in Baja California. However, because individuals who did not return for ST interpretation differed from those who completed follow-up in terms of age, sex, and locality, differential loss to follow-up may have influenced the observed geographic patterns. Therefore, although the locality-level associations remained significant after adjustment for age and sex, these findings should be interpreted cautiously as exploratory spatial exposure patterns rather than definitive geographic risk estimates.

Taken together, these findings indicate heterogeneous coccidioidin-ST+ across the sampled localities. Because the environmental analyses were based on only nine localities and no continuous environmental variable remained statistically significant after multiple-testing correction, the study cannot determine whether environmental factors explain the observed geographic differences. Future studies with larger numbers of localities, probabilistic sampling, and multivariable environmental designs are needed to evaluate this question.

### Limitations

Because participants were recruited through community-based nonprobability sampling strategies that differed across localities, some degree of selection bias was likely unavoidable. Recruitment through local health institutions may have preferentially included specific demographic or occupational groups depending on local participation dynamics. In addition, several localities had relatively small sample sizes, which may have contributed to unstable odds ratio estimates and wide confidence intervals. Similarly, some descriptive categories presented in Table 1 included small subgroup sizes, and high proportions observed in these groups should be interpreted cautiously. Accordingly, the observed locality-level differences should be viewed as exploratory exposure patterns rather than population-representative prevalence estimates for Baja California. Nevertheless, the findings provide valuable insights into regional exposure patterns within the participating communities and align with the study’s exploratory objectives. Future studies using probabilistic sampling designs and geographically balanced recruitment strategies would help improve representativeness and strengthen inference regarding regional exposure patterns.

An additional limitation concerns loss to follow-up for ST interpretation. Of the 521 individuals who received coccidioidin-ST, only 416 returned for interpretation between 48 and 72 h after application. Because positivity could only be determined among participants who returned, differential loss to follow-up may have influenced the estimated positivity. In addition, coccidioidin-ST has inherent diagnostic limitations, including the possibility of false-positive reactions related to antigenic cross-reactivity with other fungal organisms and false-negative reactions among individuals with impaired cellular immunity or receiving immunosuppressive therapy. Although antigenic cross-reactivity cannot be completely excluded, coccidioidin-ST has historically shown relatively high specificity for prior sensitization to *Coccidioides* spp. Furthermore, coccidioidin-ST reflects prior sensitization to *Coccidioides* antigens but cannot determine the timing, duration, or geographic source of previous exposure [35].

Housing characteristics were not collected as part of the questionnaire and therefore could not be evaluated in the present analysis. However, previous subnational studies conducted in Mexico [9,10] reported coccidioidin-ST+ among individuals living in both formal and informal housing settings, including urban and rural areas. In addition, several of the studied localities are situated within semi-arid environments where airborne dust exposure and soil disturbance may contribute to environmental exposure to *Coccidioides*. Nevertheless, because housing-related variables were not directly assessed, their potential contribution to exposure could not be evaluated in this study and should be considered in future investigations.

Environmental analysis also has important limitations. Although 24 environmental variables were evaluated, these predictors were summarized at the locality level, resulting in a small number of environmental units. Therefore, the Mann–Whitney U tests were used as exploratory comparisons rather than as confirmatory analyses of independent environmental effects. To reduce the risk of false-positive findings, we applied the Benjamini–Hochberg false discovery rate correction. After this adjustment, none of the evaluated continuous environmental variables remained statistically significant. Future studies including a larger number of localities are needed to fit robust multivariable environmental models and to disentangle the independent and combined effects of climate, topography, soil, and land cover.

The travel history variable was assessed as a simplified dichotomous variable and did not capture duration, frequency, timing, or environmental characteristics associated with travel-related exposure. Finally, the prolonged recruitment period (2016–2022) may have introduced temporal heterogeneity related to environmental conditions, recruitment patterns, and healthcare access. Therefore, statistically significant differences between collection years should be interpreted cautiously, as they may reflect variations in recruitment dynamics rather than true temporal differences in exposure.

## 5. Conclusions

This study identified coccidioidin-ST+ in 31.9% of sampled participants across nine localities in Baja California, with variability in positivity across the studied communities. Coccidioidin-ST+ was observed in all sampled areas, supporting the need for broader epidemiologic surveillance and increased clinical awareness of coccidioidomycosis in the region.

Exploratory locality-level analyses showed geographic variability in coccidioidin-ST+. Some high-positivity localities were located in semi-arid areas with intermediate elevations and relatively greater precipitation; however, these environmental observations were based on only nine localities, and none of the evaluated continuous environmental variables remained statistically significant after multiple-testing correction. Therefore, these findings should be interpreted cautiously as exploratory ecological patterns rather than robust environmental associations.

Although the nonprobability sampling design limits generalizability, the study provides preliminary information regarding exposure patterns in understudied regions of Baja California. Future studies using probabilistic sampling designs, geographically balanced recruitment strategies, larger numbers of localities, and multivariable environmental approaches are needed to better characterize transmission dynamics and evaluate potential environmental and social factors associated with exposure to *Coccidioides* in the region.

## Figures and Tables

**Table 1:** Sociodemographic and clinical variables according to coccidioidin skin test positivity in Baja California, Mexico (n = 416).

Variable	Category	Total, n (%)	Coccidioidin-ST+, n/N (%)	*p*-Value
Sex	Female	261 (62.7)	83/261 (31.8)	0.888
	Male	154 (37.0)	50/154 (32.5)	
Age group	≤10 y	16 (3.8)	3/16 (18.8)	0.066
	11–18 y	20 (4.8)	8/20 (40.0)	
	19–30 y	106 (25.5)	27/106 (25.5)	
	31–40 y	79 (19.0)	32/79 (40.5)	
	41–50 y	64 (15.4)	22/64 (34.4)	
	51–60 y	55 (13.2)	24/55 (43.6)	
	>61 y	30 (7.2)	6/30 (20.0)	
	No response	46 (11.0)	11/46 (23.9)	
Underlying disease	Yes	133 (32.0)	36/133 (27.1)	0.233
	No	283 (68.0)	97/283 (34.3)	
Recent respiratory illness (<3 months)	Yes	78 (18.8)	39/78 (50.0)	0.507
	No	338 (81.2)	94/338 (27.8)	
Allergies	Yes	9 (2.2)	9/9 (100)	<0.001
	No or unknown	407 (97.8)	124/407 (30.5)	
Travel to Sonora/California/Arizona	Yes	11 (2.6)	11/11 (100)	0.273
	No	388 (93.3)	109/388 (28.1)	
	No response	17 (4.1)	13/17 (76.5)	
Occupation	Homemaker (household work)	93 (22.4)	47/93 (50.5)	0.001
	Education worker	83 (20.0)	22/83 (26.5)	
	Agricultural laborer/factory worker	54 (13.0)	17/54 (31.5)	
	Office worker	40 (9.6)	10/40 (25.0)	
	Health worker	58 (13.9)	9/58 (15.5)	
	Retail/commerce worker	23 (5.5)	8/23 (34.8)	
	Field work	9 (2.2)	2/9 (22.2)	
	No response	56 (13.5)	18/56 (32.1)	
Collection period	2016	10 (2.4)	6/10 (60.0)	0.001
	2017	53 (12.7)	29/53 (54.7)	
	2018	84 (20.2)	22/84 (26.2)	
	2019	111 (26.6)	37/111 (33.3)	
	2020	136 (32.7)	32/136 (23.5)	
	2021	11 (2.6)	2/11 (18.2)	
	2022	11 (2.6)	5/11 (45.5)	

ST = skin test; coccidioidin-ST+ = positive intradermal reaction to coccidioidin; percentages in the coccidioidin-ST+ column represent the proportion of positive ST within each subgroup category; y = years of age; *p* < 0.05 indicates statistical significance.

**Table 2:** Coccidioidin skin test positivity and associated odds ratios by locality in Baja California, Mexico, 2016–2022 (n = 416).

Locality	Total, n (%)	Coccidiodin-ST+, n (%)	OR (95% CI)	*p*-Value	Positivity Group
San Felipe	30 (7.21)	5 (16.67)	Referent	—	Low
Ensenada	11 (2.64)	2 (18.18)	1.11 (0.18–6.78)	0.909	Low
Vicente Guerrero	74 (17.79)	17 (22.97)	1.49 (0.49–4.49)	0.477	Low
Terrazas del Valle	47 (11.30)	10 (21.28)	1.35 (0.41–4.43)	0.619	Low
Mexicali	106 (25.48)	27 (25.47)	1.71 (0.60–4.91)	0.319	Low
Plan Libertador	37 (8.89)	12 (32.43)	2.40 (0.74–7.82)	0.146	High
Capilla Divino Niño	11 (2.64)	5 (45.45)	4.17 (0.91–19.17)	0.067	High
La Morita	37 (8.89)	20 (54.05)	5.88 (1.85–18.72)	0.003	High
Valle de las Palmas	63 (15.14)	35 (55.56)	6.25 (2.12–18.43)	0.001	High

Coccidioidin-ST+ = positive intradermal reaction to coccidioidin; OR = odds ratio; CI = confidence interval; *p* < 0.05 indicates statistical significance; referent = reference category; — = not applicable. Low-positivity (≤30%) and high-positivity (>30%) groups were defined for comparative purposes based on a prior subnational survey in Mexico [10].

## Data Availability

The data presented in this study are available on request from the corresponding author. The data are not publicly available due to privacy and ethical restrictions.

## Supplementary Materials

The following supporting information can be downloaded at: https://www.mdpi.com/article/10.3390/jof12060407/s1, Table S1: Multivariable logistic regression model for factors associated with coccidioidin-ST positivity in Baja California, Mexico (n = 369); Table S2: Comparison of environmental variables between high- and low-positivity localities using Mann–Whitney U tests with Benjamini–Hochberg false discovery rate correction; Table S3: Distribution of abiotic variables across the nine studied locations in Baja California, Mexico.

## Abbreviations

The following abbreviations are used in this manuscript:

CM	Coccidioidomycosis
ST	Skin test
coccidioidin-ST+	Coccidioidin-positive skin test
OR	Odds ratio
CI	Confidence interval
SD	Standard deviation
°C	Degrees Celsius
mm	Millimeters
m	Meters
ENSO	El Niño–Southern Oscillation
BSks	Cold semi-arid climate (Köppen–Geiger classification)
